# Assigning channel weights using an attention mechanism: an EEG interpolation algorithm

**DOI:** 10.3389/fnins.2023.1251677

**Published:** 2023-09-21

**Authors:** Renjie Liu, Zaijun Wang, Jiang Qiu, Xue Wang

**Affiliations:** Key Laboratory of Flight Techniques and Flight Safety Research Base, Civil Aviation Flight University of China, Guanghan, China

**Keywords:** EEG, attention mechanism, bad channel interpolation, reconstruction, channel masking, interpolation algorithm

## Abstract

During the acquisition of electroencephalographic (EEG) signals, various factors can influence the data and lead to the presence of one or multiple bad channels. Bad channel interpolation is the use of good channels data to reconstruct bad channel, thereby maintaining the original dimensions of the data for subsequent analysis tasks. The mainstream interpolation algorithm assigns weights to channels based on the physical distance of the electrodes and does not take into account the effect of physiological factors on the EEG signal. The algorithm proposed in this study utilizes an attention mechanism to allocate channel weights (AMACW). The model gets the correlation among channels by learning from good channel data. Interpolation assigns weights based on learned correlations without the need for electrode location information, solving the difficulty that traditional methods cannot interpolate bad channels at unknown locations. To avoid an overly concentrated weight distribution of the model when generating data, we designed the channel masking (CM). This method spreads attention and allows the model to utilize data from multiple channels. We evaluate the reconstruction performance of the model using EEG data with 1 to 5 bad channels. With EEGLAB’s interpolation method as a performance reference, tests have shown that the AMACW models can effectively reconstruct bad channels.

## 1. Introduction

EEG through the measurement of electrical potentials on the scalp, can reflect the activity of different brain regions and plays a crucial role in understanding brain function (Zaitcev et al., 2017; Bhavsar, 2019). As the cost of EEG equipment decreases and data acquisition becomes easier, the application of EEG is becoming more widespread (Soufineyestani et al., 2020). When recording EEG signals, various factors (such as broken wire contacts, other malfunctions, bridged electrodes and abnormal impedance, etc.) can lead to electrodes failing to accurately capture the physiological information of brain neurons. These abnormal channels are commonly referred to as “bad channels” in the literature. Directly removing bad channels will reduce the number of channels and consequently alter the data dimensions (channels*times) (Hu and Zhang, 2019).

In recent years, there are more and more researchers applying machine learning techniques to EEG analysis (Lotte et al., 2018), including various applications such as fatigue detection in drivers (Zeng et al., 2018; Zhong et al., 2022), motor imagery (Al-Saegh et al., 2021; Khademi et al., 2022), sleep monitoring (Hussain et al., 2022; Phan et al., 2023), and emotion recognition (Wang et al., 2014; Xue et al., 2020; Islam et al., 2021). However, missing data caused by bad channels can significantly reduce the accuracy of machine learning models (Emmanuel et al., 2021).

The volume conduction phenomenon provides researchers with the opportunity to reconstruct bad channels by utilizing information from the unaffected channels, ensuring that the original data dimensionality is preserved. The interpolation is faced with the essential question of which channels of data to use and what proportion of each channel to use. The prevailing method used for channel interpolation is the inverse distance method, which assigns weights based on the distances among the good channels and the bad channel. Channels that are farther away are given smaller weight proportions, while closer channels are given larger weight proportions. Relying solely on the physical distance to determine weights is insufficient and fails to consider the influence of physiological factors [e.g., skull conductivity and scalp conductivity (Ollikainen et al., 1999)] on EEG signal. Measuring the impact of these physiological factors on signal transmission is challenging. However, employing data-driven models circumvents the complex analysis process. Data-driven models focus on patterns rather than the underlying causes of data changes. Each factor is treated as a variable influencing correlation, and the resulting weights stem from the correlations between these variables. Deep learning, a pivotal component of data-driven models, excels in processing high-dimensional data (LeCun et al., 2015).

This study proposes interpolation algorithms that use the attention mechanism to assign weights. The AMACW adopts a multidimensional vector representation for each channel, capturing the interrelationships among channels within a high-dimensional space. By utilizing attention mechanisms, the algorithm calculates correlations among these vectors and subsequently transforms these correlations into channel weights. During model training, CM was employed to disperse attention, resulting in the dispersion of weights. This facilitates the utilization of data from multiple channels during data generation, thereby enhancing the model’s anti-interference ability.

In recent years, the popular Transformer series models as an significant branch of the attention mechanism models, most of its models combine Softmax with the attention mechanism. The analysis found a negative correlation between some of the channels (Jackson and Bolger, 2014). The application of the Softmax function led to an inability of the model to effectively harness data from these negatively correlated channels. In order to use more channels of data in interpolation, this study introduced a simple function to replace Softmax.

AMACW has the advantage of being able to reconstruct bad channels at unknown locations. In certain open source datasets, experimenters have placed electrodes that are not part of the standard system according to the task, and the positional information of these electrodes has not been disclosed (Zheng et al., 2019; Guillot and Guillot, 2022). Consequently, when these channels are deemed as bad channels, the inverse distance method cannot be used for reconstruction. The AMACW algorithm offers an advantage in such cases as it does not depend on the positional information of the electrodes. It only requires training the model using the data from the dataset that does not contain bad channels. Once the model is trained, it can be applied to the reconstruction process.

## 2. Related research

### 2.1. Based on the distance interpolation algorithm

The distance-based interpolation algorithm, with the inverse distance method at its core, aims to accurately calculate the distances among electrodes to allocate weights more effectively.

Soong et al. (1993) evaluated the reconstruction performance of nearest-neighbor interpolation (NNI, only focuses on a few channels close to the bad channel, and each channel is given the same weight), planar-spline interpolation (PSI, projects the electrode positions onto a plane and then calculates the weights by the inverse distance method), and spherical- spline interpolation (SSI, projects the electrode positions onto the sphere and then calculates the weights by the inverse distance method). They found that the reconstruction results obtained using global interpolation methods (PSI and SSI) were superior to those achieved using local interpolation methods (NNI).

Courellis et al. (2016) expanded on the SSI method by introducing a weight allocation tactic. In their study, they incorporated three different distance measures: Euclidean distance (EuD), great-circle distance (GCD), and ellipsoidal geodesic length (EGL) to represent the distances among channels. These distance measures were utilized in conjunction with the inverse distance method to allocate weights. The researchers evaluated the performance of the model in the presence of one bad channel in the data. They found that the EGL distance measure yielded a higher reconstruction accuracy compared to the other distance measures.

Dong et al. (2021) introduced a novel model based on the three-concentric spheres head model (which provides a more accurate representation of electrode locations). By analyzing the impact of scalp conductivity, they developed a head model which is more applicable for EEG interpolation. After projecting the electrodes onto this model, the channel weights are then calculated.

### 2.2. Algorithm for fusing time information

EEG signal as a temporal signal, some scholars have studied the pattern of change in the temporal dimension of the data and applied it to data reconstruction.

Bahador et al. (2021) proposed an interpolation method that consists of two steps. (1) The data is sliced into smaller segments in the temporal dimension and each segment is interpolated using good channel data. The interpolation calculates the channel weights according to the Eud among channels. (2) Correlation of the temporal dimension. Correlations among small segments are calculated using data from the good channels, and the correlations are then converted into weights. Each small segment fuses data from other small segments by weight. Finally, the data from the small segments are stitched together to form the complete interpolated data.

Saba-Sadiya et al. (2020) designed a depth encoder-decoder model (ED_model) for bad channel reconstruction that does not calculate the distance among electrodes and thus does not require accurate localization information. The model is based on CNN, which is widely used in the image field and is improved so that it can be applied to the reconstruction of EEG data. The data processing section projects the electrode positions from three-dimensional space to a two-dimensional plane but only uses an 8*8 square grid to represent the position relationships among them. The EEG data is transformed into picture data similar to multi color channels, with the planar representing the location information among channels and the color channel of the original image becoming the temporal dimension of the EEG data.

### 2.3. Virtual EEG channel

High-density placement of electrodes can obtain more detailed information, but in practice, it leads to a lower user experience, a longer time to place electrodes and expensive costs (Sun et al., 2023). Therefore scholars have begun to investigate the use of low-density EEG to generate high-density EEG. This is similar to bad channel interpolation in that both generate new channel data based on already known channel data.

Li et al. (2022) designed the GAN (IWGAN) for EEG channel data generation using WGAN as a framework. WGAN is more stable than the original GAN (Arjovsky et al., 2017), and IWGAN further improves the training stability by improving the loss function. IWGAN generates new channel data not only similar to the real values but also incorporates other channel data. Through testing, it is found that (1) the lack of channel data decreases the accuracy of the classification task a lot, and (2) replacing the original channel data with the generated data, the accuracy is similar. This shows that for the downstream task, the generated channel data can replace the original channel data to some extent, thus reducing the number of real electrodes placed in the experiment.

Svantesson et al. (2021) use the transposed convolution in CNN to implement upsampling (Up_CNN) into more data. The model first convolves the data, encoding it from the spatial dimension to the temporal dimension. Subsequently, the data undergoes transposed convolutional decoding to revert from the temporal dimension. After decoding, the missing positions are filled in. Using the same architecture, they trained two virtual channel generation models and a bad channel interpolation model. The CNN network analyses every data so that bad channels, if any, can be detected and repaired.

Sun et al. (2023) proposed the EC-informer model based on informer (Zhou et al., 2021), which is a variant of Transformer that uses historical data to predict data in the future over a long period of time, while EC-informer uses historical data to generate data for the same time period. EC-informer uses historical data to generate data for the same time period. EC-informer inherits the low computational complexity of informer, but at the same time has fewer input channels (4 channels) than other EEG generation models, resulting in less computational effort in the model as a whole, thus reducing the computational load on the EEG processing equipment. This is especially important for portable BCI devices.

### 2.4. Attention mechanisms

When a substantial amount of information is presented, humans tend to selectively attend to a portion of the information while disregarding other components (Buschman and Miller, 2010). Similarly, attention mechanisms in deep learning serve a comparable purpose, allowing models to process data without being restricted by positional constraints (Dosovitskiy et al., 2021) or sequential order (Vaswani et al., 2017). These models have the capacity to autonomously select relevant and significant information based on the task.

In recent years, the attention mechanism has attracted more and more attention in the field of deep learning, and the application areas have been expanded from natural language processing to the image field. Vaswan et al. proposed the first transformer model using only the attention mechanism in “attention is all you need,” and the model and its variants have achieved excellent results in the field of natural language processing. This model and its variants have achieved excellent results in the field of natural language processing. The transformer model has been continuously improved to set new records in several tasks, and Alexey et al. and Liu et al. believe that the attention mechanism is also applicable to the image domain, and they have improved the transformer model and applied it to several image tasks. Informer and EC-informer are both centered on the attention mechanism. However, compared with the AMACW model proposed in this study, there is a big difference in the model structure and the data processing process. The EC-Informer structure encodes the EEG data and sends it into the multi-layer attention structure for computation, which leads to a huge increase in the computational load of the model. The EEG data in the AMACW algorithm will only operate with the results of the attention calculation, reducing the amount of computation.

## 3. Methods

Interpolation methods based on distances range from PSI (2 Dimensions), spherical interpolation (3D), to three-concentric sphere (3D). Models representing the relationship between electrodes increase in model dimensions as more factors are considered. The AMCMW algorithm proposed in this study uses an Embedding layer to represent the relationship between channels in a high dimensional space. The attention mechanism is then used to describe, the correlation between the channels and then the correlation is transformed into interpolated weights.

**Figure 1:**
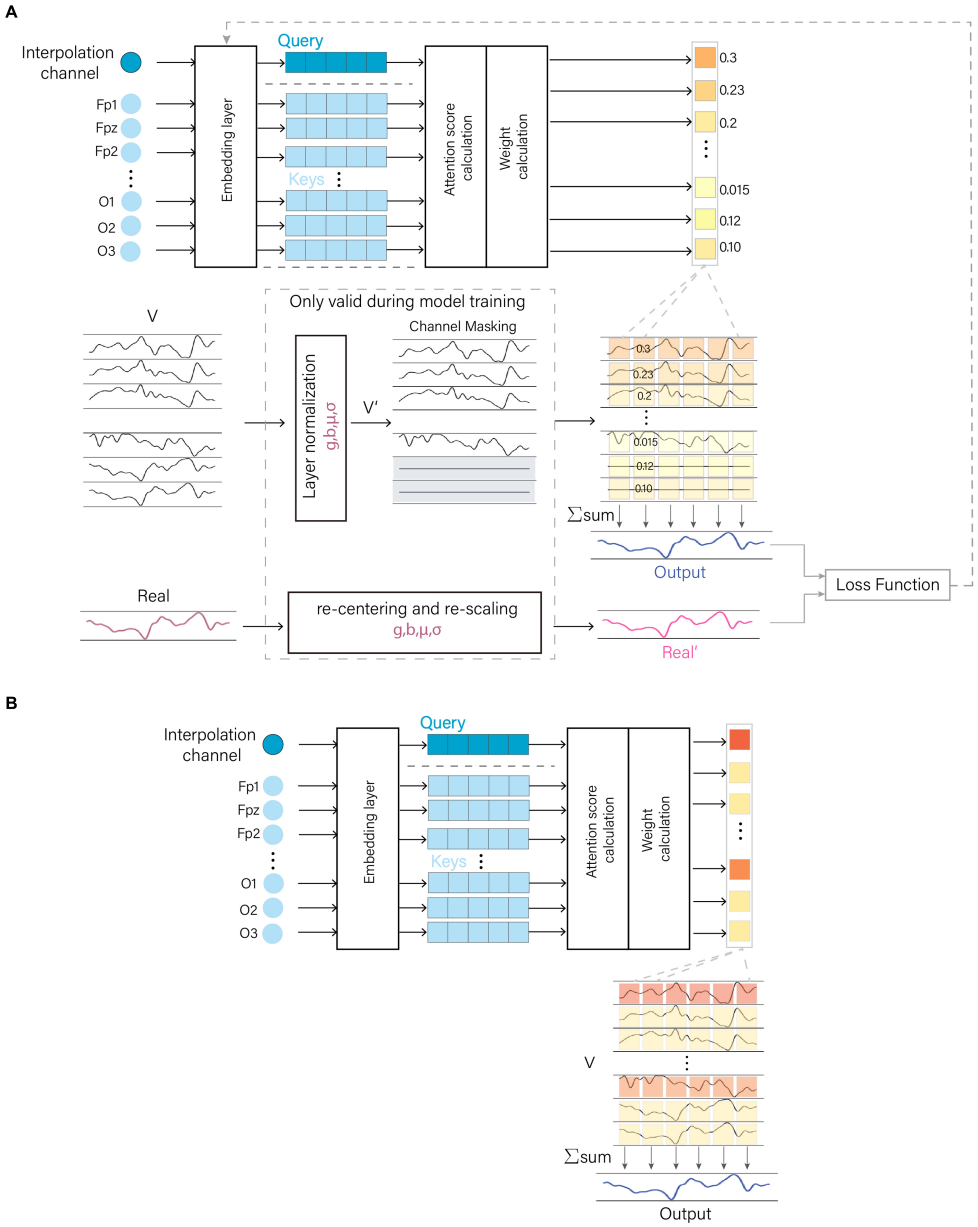
AMACW algorithm. The channel names are converted to vectors by the embedding layer, and then the weights of each channel are obtained by the attention score calculation layer and the weight calculation layer. In the training stage, the original data is optimally distributed by Layer Normalization (LN), and then a random part of channels are zeroed in the channel masking layer (CM). The processed data are summed by weights to get the output data. In order to minimize the difference between the distribution of the real values and that of the input data, the real values are subjected to the same operations as in the LN layer. CM and LN are valid only in the training stage. **(A)** Model training stage and **(B)** Model testing stage.

The AMACW consists of a channel embedding layer, an attention computation layer, a weight calculation layer and output layer. The embedding layer transforms the channel names (or order) into feature vectors. The attention computation layer utilizes these feature vectors to calculate the correlations between the bad channel and other channels, and the weight calculation layer transforms these correlations into weights. Original EEG data was passed through a layer normalization to optimize the data distribution. The processed data are randomly zeroed for some channels in the channel mask layer. During training, the model employs a CM to optimize the allocation of attention, thereby enhancing the model’s robustness and interference resistance. The structure of the AMACW algorithm model and the CM is depicted in Figure 1.

### 3.1. Channel-embedding layer

For the model to take into account the many factors that affect the EEG signal, we propose channel embedding. Channel embedding is the representation of each channel as a high-dimensional vector. These feature vectors are variables and the training process of the model can be understood as the vectors finding the right position in space to represent themselves in relation to other channels. Distance-based algorithms only represent correlations between channels in 3D space, using a channel embedding layer allows for a more detailed and comprehensive description of the factors thus improving interpolation accuracy.

The dimension of vectors impacts the performance of models. If the dimension is too small, it may not capture the complex relationships among channels, while a dimension that is too large can lead to issues such as overfitting and longer training time (Yin and Shen, 2018). Regarding the vector dimension of the channel-embedding layer, two factors need to be taken into account: (1) there are many physiological factors that influence EEG (Ollikainen et al., 1999), and more variables can describe these influences in more detail. (2) the number of training samples is limited, and too many variables increase the risk of overfitting. Therefore, this study devises two models with vector dimensions of 20 and 35 and evaluates their performance.

### 3.2. Attention computation layer

Attention computation involves matching the task vector with the background vectors using a score function to determine the distribution of attention (Niu et al., 2021). The feature vector of the bad channels (query) and the feature vector of other channels (key) calculate the similarity separately to obtain attention scores. AMACW employs the dot_product (Luong et al., 2015) as the matching method, which is a global attention computation approach. The attention scores were computed as:


$$\mathrm{score}\;\left(\mathrm{query,keys}\right)=\mathrm{query}*{\mathrm{keys}}^{T}$$

The key characteristic of dot_product is that the query is compared with each key to calculate their similarity, enabling the model to observe all channels.

### 3.3. Weight calculation layer

The attention scores are transformed into weights for each channel, which is similar to the normalization layer in classification models, which converts inputs into proportions. The choice of a normalization function that matches the task can accelerate model convergence and improve accuracy (Martins et al., 2016; Zhang and Sennrich, 2019).

**Figure 2:**
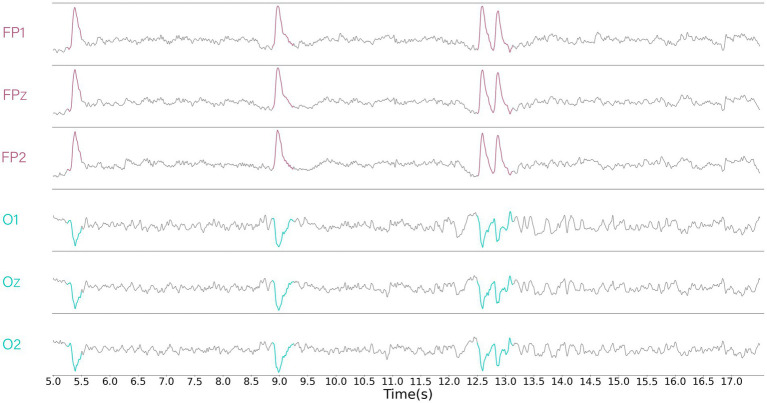
Displays the data from part of the channels, revealing segments that exhibit obvious negative correlations. It can be clearly observed that the red region is negatively correlated with the blue region.

Visualizing the EEG signals, it can be found that some of the data between the EEG channels are negatively correlated (e.g., Figure 2). Although the use of the Softmax function as a weight calculation function was able to suppress the expression of channels that were poorly correlated, it suppressed the expression of channels that were negatively correlated. Therefore, we have chosen a simple and commonly used proportional calculation method that retains attention on negatively correlated channels and yields negative weights. The Simple function to calculate the proportion of each component to the sum of the absolute values is:


$$\mathrm{weight}=simple\left(score\right)=\frac{score_{i}}{\sum_{j=1}^{N}\left|score_{j}\right|}\times 100\%$$

### 3.4. Output layer

Layer Normalization (LN) is widely applied to the Transformer family of models, the benefit of accommodating variable input data lengths. That is, the length of data processed by the model is not fixed (e.g., for training, the length of each input data is 200, and for testing, the length of each input data is 2000). Although the functioning principle of LN is still unclear, through a large number of experiments and analyses, it is generally agreed that normalization can smooth the gradient, accelerate model convergence and improve generalization ability (Ba et al., 2016; Xu et al., 2019).

During the model training stage, good channel data are adjusted to the data distribution through the normalization. Models will not use LN in testing. LN is calculated as follows:


$$V^{\prime }=\frac{g}{\sigma }⊙\left(V-\mu \right)+b\mu =\frac{1}{H}\overset{H}{\underset{i=1}{\sum }}V^{i}\sigma =\sqrt{\frac{1}{H}}\overset{H}{\underset{i=1}{\sum }}\left(V^{i}-\mu \right).$$

Where *g* and *b* are learnable parameters denoting gain and bias. ⊙ is a dot production operation. *μ* and *σ* denote the mean and variance of the good channel data. *H* is the number of input data per round of training. *v* is the good channel EEG data.

The same operation is applied to the real data (label data) to reduce the difference between the values of the input data and the label data. The calculation is as follows:


Real′=gσ⊙(Real−μ)+b

Where the values of *g*, *b*, *μ*, and *σ* are the same as in the LN.

After calculating the weights for each channel, the normalized data is summed by weight to generate the interpolated data.


$$Interpolation=\overset{N}{\underset{j=1}{\sum }}weight_{j}\times V_{j}^{\prime }$$

### 3.5. Channel masking layer

In the initial training, it had been found that attention would have been focused on one channel that had been close to the bad channel in physical space. This resulted in interpolation with data having come from almost this one channel, which could have led to large reconstruction errors if the data in this channel had been disturbed. Therefore we have designed an EEG channel masking (CM) method. CM effectively spread attention, thereby reducing the influence of data abnormalities from a few channels on the process of data reconstruction.

CM is a process of randomly zeroing some of the channel data before it is fed into the model. If the model allocates a large amount of attention to the masked channel, it will cause the output to tend to zero, resulting in a particularly large loss value and forcing the model to adjust its parameters to divert some attention to other channels.

**Figure 3:**
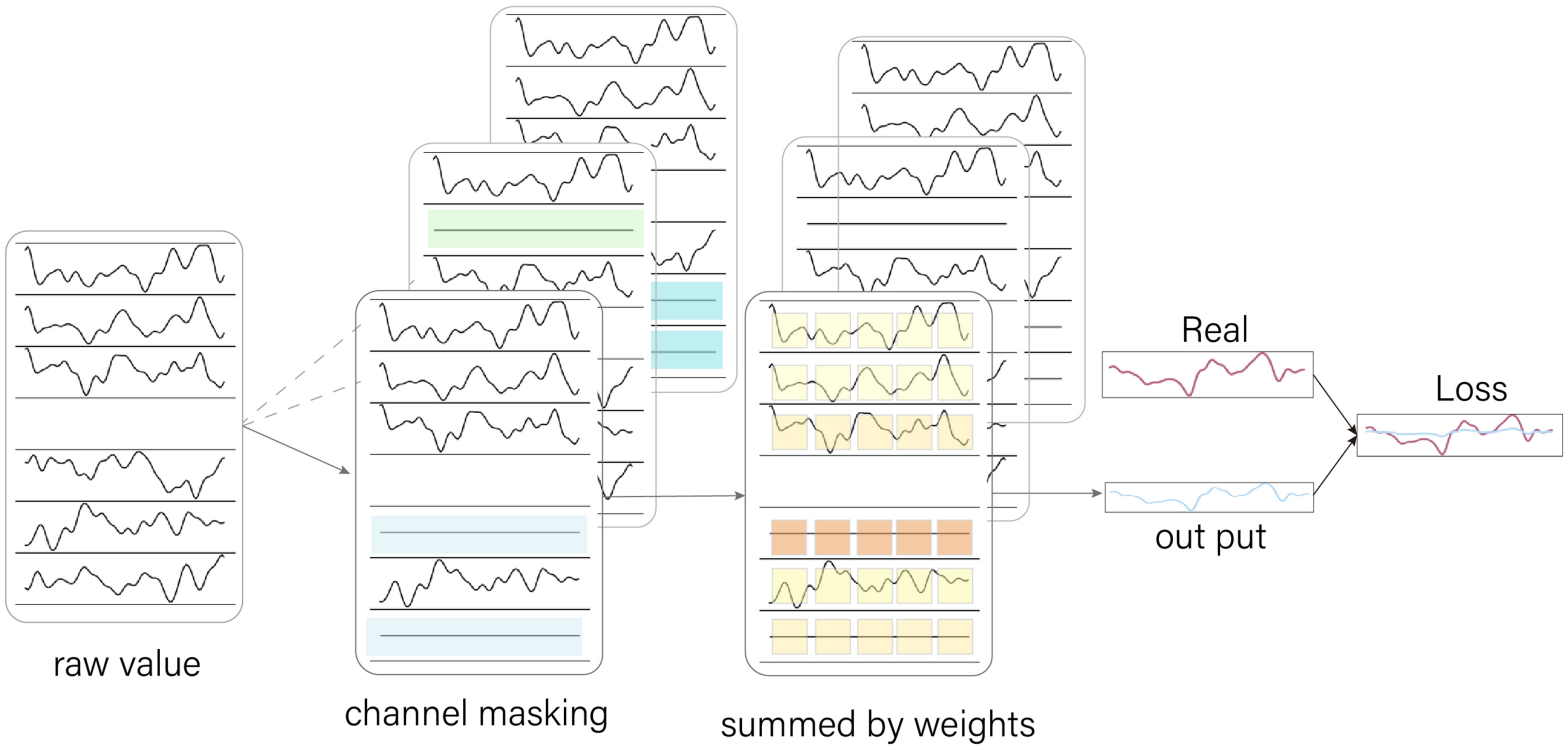
Channel Masking. The channel masking is a random zeroing of the data by 0–5 channels. Because the channels are chosen randomly, they are received differently for the model at different epoch, increasing the amount of sample data. When the model focuses too much attention on the zeroed channels, it causes the output values to converge to 0, which leads to excessive loss values, forcing the model to spread its attention.

In each training epoch, CM randomly zeroes a part of the data (Figure 3). This causes new samples to be generated from the same original sample due to the different areas of zeroing. This increases the training sample size of the model, which helps to improve the generalization ability of the model.

**Figure 4:**
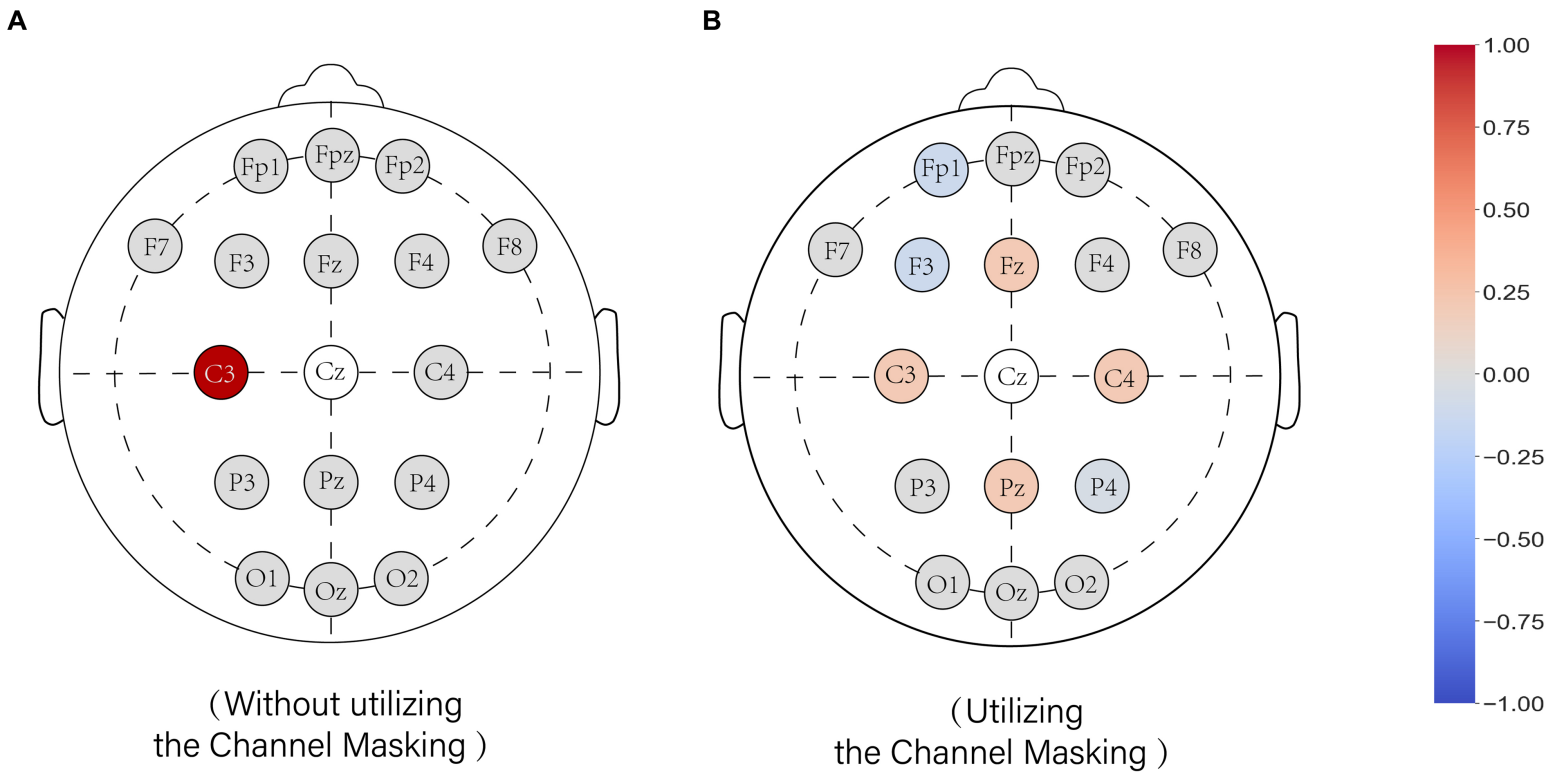
The effect of the channel mask on the weight assignment of the model. Weight values of the output model when interpolated to the CZ. **(A)** shows the model trained without the channel mask and **(B)** shows the model trained with the channel mask. The colors represent the weights for the channels, with red representing positive weights, gray representing zero, and blue representing negative weights.

Taking the reconstruction of channel CZ as an example, Figure 4 (left) illustrates the weight distribution of the model without using the CM mechanism during training, while Figure 4 (right) shows the weight distribution of the model using the CM mechanism. Without using the masking mechanism, most of the weights are allocated to channel C3. When the masking mechanism is applied, the weights are distributed among channels FZ, C3, C4, and PZ, ensuring that the weights are not excessively concentrated on a single channel.

## 4. Experimental evaluation

There are two application situations for the AMACW algorithm: (1) The electrodes in the dataset all belong to the standard system, and the model is used directly to repair the bad channels. The ACWM model is used in the same way as other models. (2) The bad channels in the dataset do not belong to the standard lead system and their location information is unknown. Traditional location-based algorithms are unable to perform bad channel repair. The AMACW algorithm can train a proprietary model using the good channels data in the dataset. Considering that in general, the amount of data captured by EEG experiments is limited, we used only a small amount of data to train the model. For convenience of description later in the text. The model applied to the first case will be referred to as the general model and the second as the special model.

### 4.1. Data sources

If there is too much similarity in the data, this could allow the model to learn some particular features leading to overfitting. We have collected some datasets with variability with the aim of simulating real use cases and testing the robustness of the algorithm. A number of four datasets were used for the experiments, namely SEED-V (Liu et al., 2022), A test–retest resting and cognitive state EEG dataset (TRCS) (Wang et al., 2022), EEG Motor Movement/Imagery Dataset (MMI) (Schalk et al., 2004), and The OpenMIIR Dataset (MIIR) (Stober et al., 2015). These datasets were collected by different laboratories under different experimental tasks (Table 1).

**Table 1:** Brief description of the four datasets.

Dataset name	Background	Number of participants	Device	Sampling frequency
SEED-V	Watching the video induced happy, sad, fearful, disgusted, and neutral emotions in participants	20	Neroscan	1,000 HZ
A test–retest resting and cognitive state EEG dataset(TRCS)	Resting (eyes-open and eyes-closed) and subject-driven cognitive states (memory, music, subtraction)	60	Brain Products	500 HZ
EEG Motor Movement/Imagery Dataset(MMI)	Participants perform 4 tasks based on on-screen prompts. The tasks included imagined movements and real movements	109	Not mentioned in the original article	160 HZ
The OpenMIIR Dataset(MIIR)	Participants listen to and visualize 12 short music clips (7 to 16 s each)	10	Not mentioned in the original article	512 HZ

SEED-V, TRCS, and MIIR show data from the entire recording process, the authors of MMI have edited the data to retain only a small amount of data from the task phase.

### 4.2. Data processing and dataset preparation

**Figure 5:**
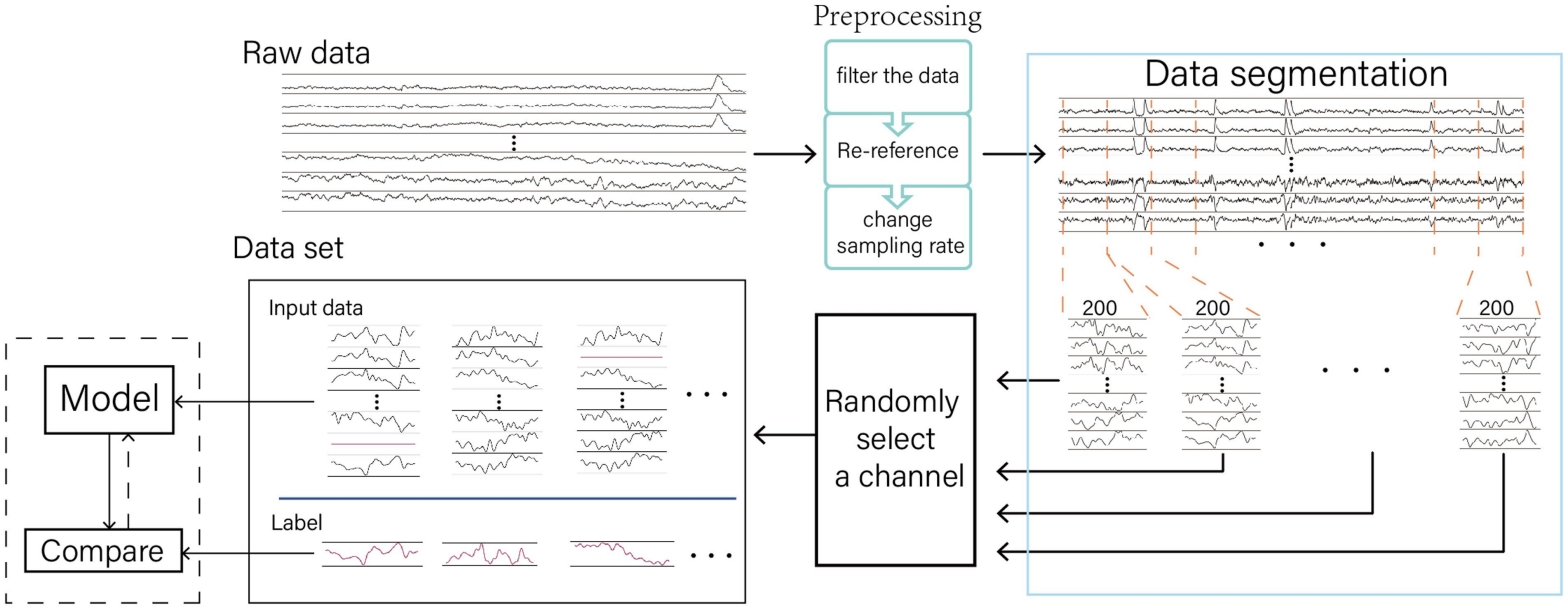
Entire data processing process. The raw data are pre-processed and cut into small segments. After randomly selecting a channel as a bad channel, the positions of the original bad channel are filled with zeros. The data values of the original bad channel are the labels for model training.

The data exported from the EEG device is simply preprocessed and then made into a dataset (the entire processing flow is shown in Figure 5). The preprocessing steps include re-referencing (average reference), filtering [0.1–30 Hz (Hu and Zhang, 2019)], and resampling (according to Nyquist’s rule, the resampling frequency is half of the sampling frequency). To ensure fairness in performance comparisons with open-source models, we retained only the channels common to both the 10–20 system and the two datasets. After preprocessing, the data is segmented into intervals of 200 data, and a random channel is selected to be set to zero, while its original value and channel name are recorded.

The general model was trained using data from participants #1–5 in SEEV-V and MIIR. It was then tested using participant #1 in MMI and participant #1 data in TRCS. The training and test data came from different datasets, which better reflects the effect of the model when it is actually used. Special models were trained using only participants #1, #2, and #3 from SEED-V and tested using participants #4 and #5 from SEED-V. The proprietary model was trained using a small amount of data because the electrodes at unknown locations were mostly special electrodes placed by the experimenter according to the task, and it was difficult to collect other datasets using the same special electrodes. And most EEG-related experiments do not collect large amounts of EEG data (Mahesh, 2018).

An increasing number of scholars are using more dense electrode placement schemes in EEG experiments (Oostenveld and Praamstra, 2001). When some disturbances (e.g., sweat) are present, the number of affected electrodes increases (Sun et al., 2023). To test the model’s redundancy, testing data with 1 to 5 bad channels were created.

**Figure 6:**
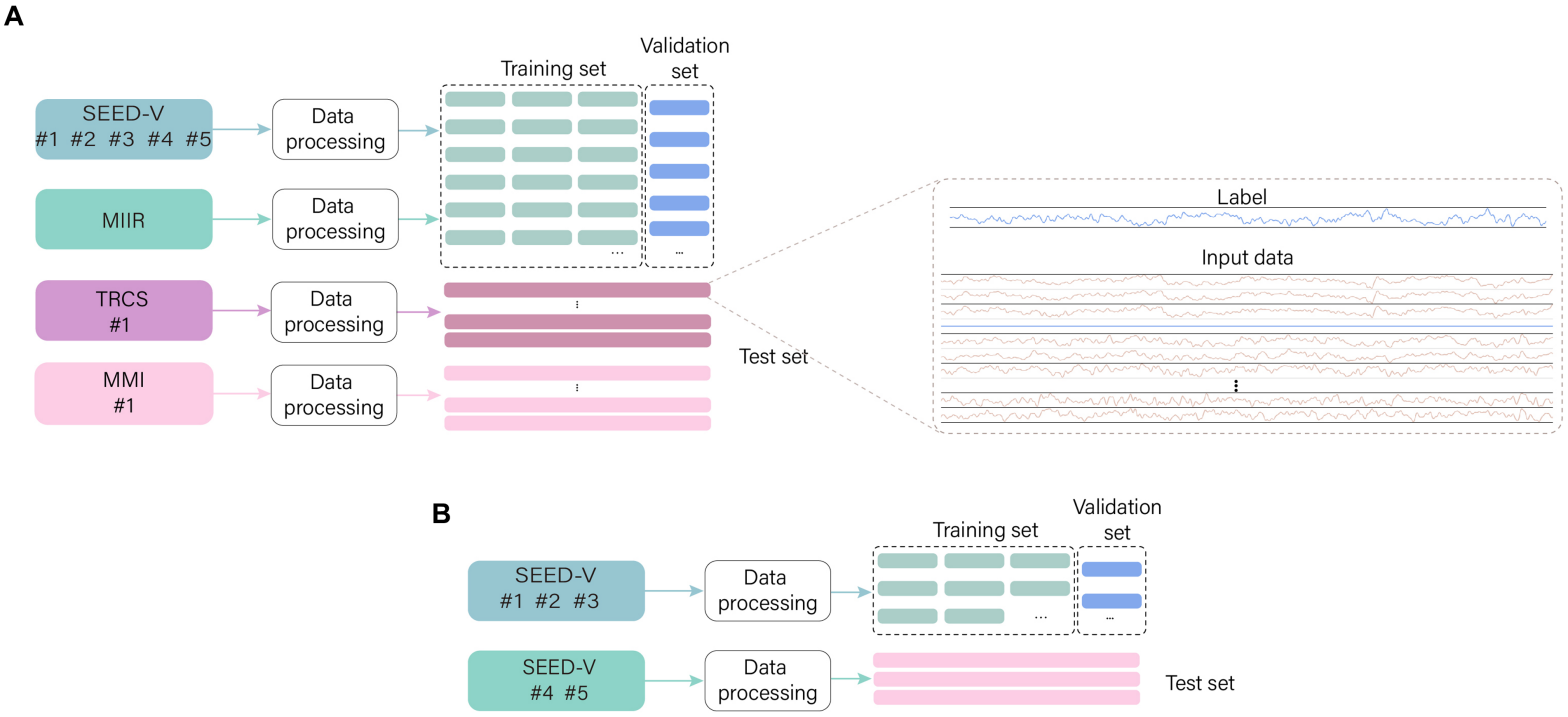
The names of different sets. The data are preprocessed in the same way, with the training data being sliced into small segments and 10% randomly selected to form the validation set. The test data is preprocessed without segmentation which facilitates testing. **(A)** General models use datasets and **(B)** Special models use datasets.

The model training is done to estimate the performance of the model, 10% of the training data is randomly selected from the training data as the validation set. The data sets for different purposes are named in order to facilitate the descriptions in the subsequent sections (Figure 6). (1) Training set: 90% of the training data. (2) Validation set: 10% of the training data. (3) Test set: test data.

### 4.3. Experiment setup

#### 4.3.1. Comparison model

EEGLAB, as a mainstream EEG data processing platform, contains two algorithms, Spherical and Planar. These two algorithms belong to the traditional distance-based interpolation methods and can be used directly without training. We take these two algorithms as the baseline.

In contrast to the deep learning approach for calculating correlation, representing inter-channel correlation using the Pearson correlation coefficient is simpler and more time-efficient. Therefore, we created a model based on PCC. Initially, within the training set, correlation coefficients among channels are computed, and then these coefficients are converted into weights for use in interpolation.

In this study, we constructed DE_model, IWGAN, and Up_CNN as mentioned in the related work section. The DE model structure is fixed (input data length and channel data amount are non-variable). The structure of IWGAN and Up_CNN can be adjusted according to the number of channels and the length of the generated data.

#### 4.3.2. AMACW model

In this study, we designed two models, namely “simple_20” (embedding dimension = 20) and “simple_35,” based on different embedding dimensionality.

These models are constructed according to the requirement of 17 channels and generating 200 number of data points per computation. The computational amount (generating 200 data points) and the number of parameters of the model are shown in Table 2. The computational amount and the number of parameters of the AMACW model are much smaller than other models, which means that the AMACW model consumes only a very small amount of computing resources and storage space, which is beneficial to be applied to wearable devices.

**Table 2:** Model generates 200 data calculations and the number of parameters of the model.

Model name	PFLOs	Parameters
Simple_20	5.8 M	374
Simple_35	6.1 M	629
ED_model	96.0 M	1.9 M
IWGAN	637.7 M	28.4 M
Up_CNN	855.5 M	517.6 M

We do not limit the training time and number of rounds for the model. For every 100 epochs of training, the validation set is used to test the training effectiveness of the model, and when the loss value of the model in the validation set only fluctuates in a small range, the model is considered to have converged and then training is stopped.

### 4.4. Results

The models are trained using data from the training set and the validation set is used to filter out the models that perform better. The models are then tested for performance on the test set.

We chose MSE to assess the performance of the model, calculated as follows:


$$MSE=\frac{\sum_{i=1}^{N}{\left(y-\hat{y}\right)}^{2}}{N}$$

Where y is the true value (original value). $\hat{y}$ is the predicted value. This is the data calculated by the model. *N* is the number of data inferred in total.

The MSE quantifies the degree of difference among the estimated values and the ground truth values. A higher value indicates a greater discrepancy among the estimated and actual values, indicating lower estimation accuracy of the model. The error value of the model for each of the bad channel conditions is the mean MSE of multiple samples: $erro=\frac{\sum_{k=1}^{M}MSE_{k}}{M}$ ,where M is number of samples.

The general models and comparison models perform in the test set as shown in Tables 3, 4. The special models are used in the background of unknown positional information. Spherical and Planar as distance based algorithms are required to obtain positional information, therefore they are interpolated with the positional information obtained first (Table 5).

**Table 3:** Error values in the MMI dataset.

		Number of bad channels					
Model name		1	2	3	4	5	Sum
AMACW	Simple 20	94.40	156.71	242.45	258.38	315.63	1067.57
	Simple 35	**90.87**	**126.26**	**130.67**	**172.28**	**208.07**	**728.15**
EEGLAB	Spherical	94.13	131.02	215.26	204.29	291.05	935.75
	Planar	292.53	402.59	548.37	664.73	679.42	2587.64
PCC		472.80	490.33	510.26	532.92	617.3	2623.61
ED_model		179.12	238.47	271.97	298.71	303.65	1291.92
Up_CNN		281.34	287.58	283.69	293.72	331.78	1478.11
IWGAN		233.29	273.52	263.52	306.83	313.81	1390.97

Bolded characters indicate the method with the lowest error value for that number of bad channels.

**Table 4:** Error values in the TRCS dataset.

		Number of bad channels					
Model name		1	2	3	4	5	Sum
AMACW	Simple 20	5.75	6.59	8.62	9.08	10.51	40.55
	Simple 35	4.55	**4.23**	**5.73**	**6.27**	**7.62**	**28.40**
EEGLAB	Spherical	**1.52**	5.18	7.15	9.93	10.04	33.82
	Planar	23.08	18.18	19.46	18.66	18.25	97.63
PCC		24.04	25.75	25.30	26.63	26.79	128.51
ED_model		7.02	8.09	8.65	8.28	9.55	41.59
Up_CNN		10.33	11.32	11.24	12.40	12.76	58.05
IWGAN		11.48	12.07	12.90	12.26	13.89	62.60

Bolded characters indicate the method with the lowest error value for that number of bad channels.

**Table 5:** Error values when the AMACW model is used as a special model.

		Number of bad channels					
Model name		1	2	3	4	5	Sum
AMACW	Simple 20	19.71	24.63	25.52	37.63	47.87	155.36
	Simple 35	17.58	23.76	**25.02**	**27.95**	**29.79**	**124.10**
EEGLAB	Spherical + location	**11.66**	**17.49**	25.53	37.65	40.54	132.87
	Planar + location	65.63	51.65	56.21	52.79	53.18	279.46

EEGLAB’s algorithm is reconstructed after obtaining information on the electrode positions. Bolded characters indicate the method with the lowest error value for that number of bad channels.

The disparity in error values across all models in the two test sets is substantial. Consequently, we focus more on the performance comparison of different models in the same test set. In the TRCS dataset, Spherical algorithm has higher reconstruction accuracy than AMACW model in the presence of 1 bad channel. However, as the number of bad channels increases, the error value of the AMACW model grows slowly. Simple35 reduces the error by 22.06% compared to Spherical in the presence of 3 bad channels. The error values of the generative model for virtual channels are almost unaffected by the increase in channels. In general, AMACW model has higher reconstruction accuracy with multiple bad channels.

The reconstruction accuracy of the special model for a small number of bad channels (1–2 bad channels) is lower than that of the Spherical but higher than that of the Planar. This indicates that the interpolation of the special model for bad channels with unknown locations is usable but not very accurate. In the presence of 3–5 bad channels, the accuracy of the special model is higher than that of Spherical. Therefore the AMACW method can be used as a way to handle the situation encountered here in experiments using open source datasets, compensating for the problem of interpolating bad channels with unknown locations by traditional methods.

### 4.5. Analysis

Visualizing the reconstructed data and the real data, we observed that at most points the reconstructed data were close to the real values. However, when several bad channels are close to each other, the interpolation algorithm fits poorly at points with higher amplitudes, and a similar phenomenon was observed in the Bahador et al. (2021) and Li et al. (2022) experiments. This part of the data points causes a large reconstruction error.

**Figure 7:**
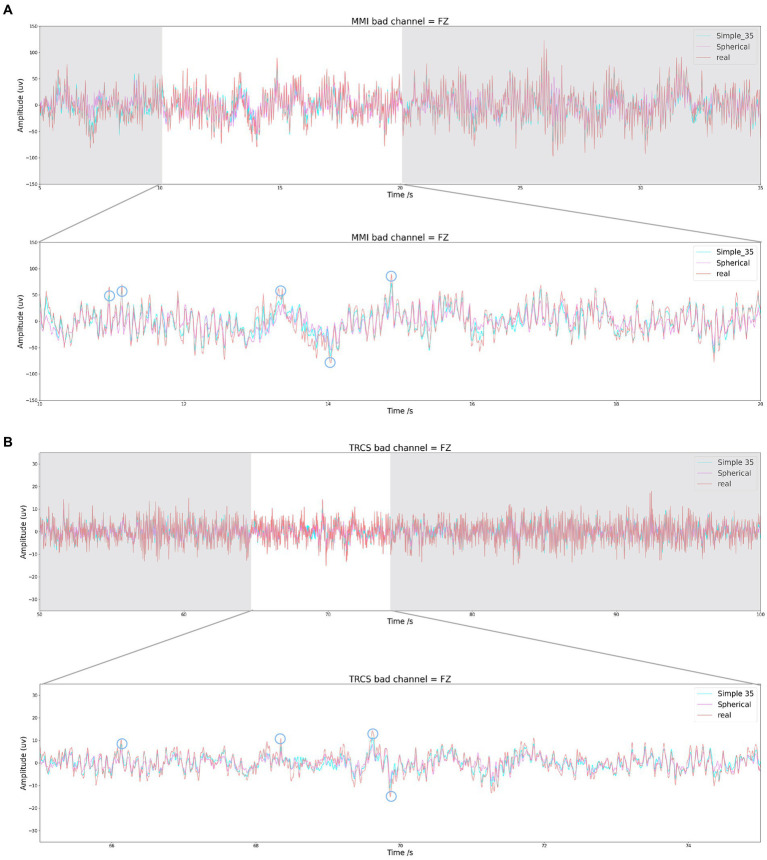
Interpolation results for Simple 35 and Spherical when FZ is bad channel. **(A)** is the MMI dataset and **(B)** is the TRCS dataset.

When both FZ and CZ are bad channels, the effect of reconstructing the FZ channel is shown in Figure 7 (subplot a is the effect of the MMI dataset and subplot b is the effect of the TRCS dataset). Both algorithms fit well at points with low amplitude, but poorly at points with higher amplitude. However, these higher magnitude points are within the normal assignment range and cannot be arbitrarily eliminated. By circling some of the points with high amplitude, it can be observed that the Simple35 model fits the points with large amplitude better than Spherical. because the algorithm fits the points with large assignment poorly and the point amplitude of the MMI dataset is significantly larger than that of the TRCS dataset, which leads to a large difference in the error value of the model in the two datasets.

The reconstruction accuracy of the AMACW model is lower in the presence of one bad channel. We analyze that this could be due to the following two reasons. (1) The limited amount of training data may lead to insufficient training of the model (Alexandropoulos et al., 2019; Song et al., 2022). The model has more learnable parameters, thus its capacity for fitting is greater. However, this also leads to an increase in the required training data and training time. (2) The training data is contaminated with excessive noise (Alexandropoulos et al., 2019; Song et al., 2022). In contrast to the data processing procedures employed by other deep learning models, the data processing approach in this study is relatively straightforward, lacking manual data filtering and denoising. Implementing a semi-automatic preprocessing step for EEG data would likely enhance data quality, offering more benefits for model training. But this increases the preparation time and training cost of model training.

The application of virtual EEG channel generation models is more biased toward large scale data generation (large ratio of generated data to original data), while the task of bad-channel interpolation belongs to small scale. For example, Up_CNN initial two models CN1,CN2 in the original paper used 4 channels to generate 14 channels (425%) and 14 channels to generate 7 channels (50%), respectively. This explains the lower accuracy of Up_CNN and IWGAN at 1–3 bad channels and the insensitivity of the error value to the number of bad channels.

**Figure 8:**
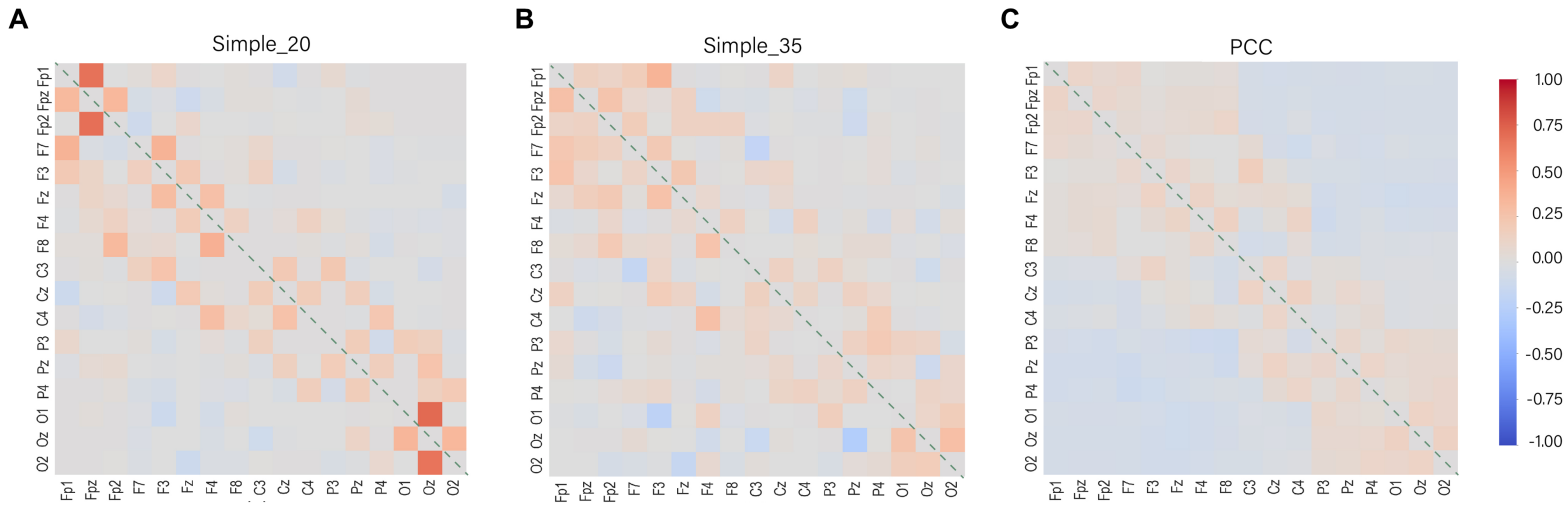
Weight distribution of the model. The diagonal line represents the weight distribution between the channel and itself, the interpolation will not do the weight distribution by itself, so it is represented by zero. The colors represent the weights for the channels, with red representing positive weights, gray representing zero, and blue representing negative weights. **(A)** Simple_20 model, **(B)** Simple_35 model and **(C)** PCC model.

The parameters that can be learned remain fixed after model training, and the distribution of model weights is visually represented (Figure 8). The weight distribution does not exhibit excessive concentration and encompasses both positive and negative values. This demonstrates that the Simple function assists the model in effectively utilizing negatively correlated channel data, enabling the model to use more channels when interpolating. The more dispersed attention of the Simple 35 (Figure 8B) compared to the Simple 25 (Figure 8A) indicates that the increased dimensionality of the channel coding vectors facilitates the model to discover more channel information. There is a difference between the two in the positive and negative weights assigned to some of the weakly interrelated channel combinations, but the absolute value of the weights is very small so it has little effect on the output results.

The weight distribution in the PCC model is highly dispersed (Figure 8C). This is attributed to its lack of suppressive capability in representing channels with weak associations. The disadvantage of weight dispersion is that data from weakly correlated channels are used even if the strongly correlated channel is the good channel is. In such cases, channels with weak correlations contribute noisy data. However, the advantage of weight dispersion is that it allows interpolation from a wider range of data sources and reduces the dependence on a single channel. This also explains the phenomenon that the PCC model does not show a significant decrease in accuracy when the number of bad channels increases.

## 5. Conclusion

The utilization of interpolation for channel data reconstruction presents an effective approach to alleviate the adverse effects caused by defective channels during subsequent analysis. The proposed AMACW algorithm in this study enables reconstruction without requiring electrode positional information. This addresses the issue of being unable to reconstruct bad channels with unknown electrode positions in certain open-source datasets. Furthermore, when positional information is available, the AMACW model trained on a small amount of data exhibits higher reconstruction accuracy for data containing multiple bad channels compared to the Spherical method in EEGLAB.

If more datasets can be collected and more computing power can be obtained, a general EEG bad channel reconstruction model can be established with a standard lead system. Diversified data for training will further improve the generalization performance and reconstruction accuracy of the model. In cases where unknown locations of bad channels occur during certain experiments, fine-tuning the model would be sufficient to reconstruct those channels.

We aspire for channel-embedding to serve as a foundation for various EEG related tasks, akin to word-embedding in NLP, thereby furnishing feature information of channels for the analysis models of EEG signals.

## Data availability statement

Publicly available datasets were analyzed in this study. This data can be found at: https://bcmi.sjtu.edu.cn/home/seed/; https://openneuro.org/datasets/ds004148/versions/1.0.1.

## Author contributions

RL conceived the model and wrote the code, wrote and translated the article, and participated in some of the data processing work. ZW was responsible for project management, and data collection, checked the code and reviewed the paper. JQ participated in some of the data processing work. XW participated in some of the data processing work and translation of the paper. All authors contributed to the article and approved the submitted version.

## Funding

This work was supported by the Independent Research Projects for Key Laboratory of Flight Techniques and Flight Safety (FZ2022ZZ02) and the Postgraduate research innovation project (X2023-32) of the Civil Aviation Flight University of China (CAFUC).

## Conflict of interest

The authors declare that the research was conducted in the absence of any commercial or financial relationships that could be construed as a potential conflict of interest.

## Publisher’s note

All claims expressed in this article are solely those of the authors and do not necessarily represent those of their affiliated organizations, or those of the publisher, the editors and the reviewers. Any product that may be evaluated in this article, or claim that may be made by its manufacturer, is not guaranteed or endorsed by the publisher.

## References

[ref1] Alexandropoulos S.-A. N. Kotsiantis S. B. Vrahatis M. N. (2019). Data preprocessing in predictive data mining. Knowl. Eng. Rev. 34:e1. doi: 10.1017/S026988891800036X

[ref2] Al-Saegh A. Dawwd S. A. Abdul-Jabbar J. M. (2021). Deep learning for motor imagery EEG-based classification: a review. Biomed. Sig. Process. Control 63:102172. doi: 10.1016/j.bspc.2020.102172

[ref3] Arjovsky M. Chintala S. Bottou L. (2017). Wasserstein GAN. Available at: http://arxiv.org/abs/1701.07875 (accessed August 21, 2023).

[ref4] Ba J. L. Kiros J. R. Hinton G. E. (2016). Layer normalization. Available at: http://arxiv.org/abs/1607.06450 (accessed August 28, 2023).

[ref5] Bahador N. Jokelainen J. Mustola S. Kortelainen J. (2021). Reconstruction of Missing Channel in EEG using spatiotemporal correlation-based averaging. J. Neural Eng. 18:056045. doi: 10.1088/1741-2552/ac23e2 34488198

[ref6] Bhavsar R. P. (2019). Assessing variability of EEG and ECG/HRV time series signals using a variety of non-linear methods.

[ref7] Buschman T. J. Miller E. K. (2010). Shifting the spotlight of attention: evidence for discrete computations in cognition. Front. Hum. Neurosci. 4:e00194. doi: 10.3389/fnhum.2010.00194, PMID: 21119775 PMC2990535

[ref8] Courellis H. S. Iversen J. R. Poizner H. Cauwenberghs G. (2016). EEG channel interpolation using ellipsoid geodesic length. In 2016 IEEE biomedical circuits and systems conference (BioCAS) (Shanghai, China: IEEE).

[ref9] Dong L. Zhao L. Zhang Y. Yu X. Li F. Li J.. (2021). Reference electrode standardization interpolation technique (RESIT): a novel interpolation method for scalp EEG. Brain Topogr. 34, 403–414. doi: 10.1007/s10548-021-00844-2, PMID: 33950323 PMC8195908

[ref10] Dosovitskiy A. Beyer L. Kolesnikov A. Weissenborn D. Zhai X. Unterthiner T.. (2021). An image is worth 16x16 words: Transformers for image recognition at scale. Available at: http://arxiv.org/abs/2010.11929 (accessed September 20, 2022).

[ref11] Emmanuel T. Maupong T. Mpoeleng D. Semong T. Mphago B. Tabona O. (2021). A survey on missing data in machine learning. J. Big Data 8:140. doi: 10.1186/s40537-021-00516-9, PMID: 34722113 PMC8549433

[ref12] Guillot A. Guillot A. (2022). Dreem-automated-sleep-staging. kaggle. Available at: https://www.kaggle.com/c/about/community.

[ref13] Hu L. Zhang Z. (2019). EEG signal processing and feature extraction. Singapore: Springer Singapore.

[ref14] Hussain I. Hossain M. A. Jany R. Bari M. A. Uddin M. Kamal A. R. M.. (2022). Quantitative evaluation of EEG-biomarkers for prediction of sleep stages. Sensors 22:3079. doi: 10.3390/s22083079, PMID: 35459064 PMC9028257

[ref15] Islam R. Islam M. Rahman M. Mondal C. Singha S. K. Ahmad M.. (2021). EEG Channel correlation based model for emotion recognition. Comput. Biol. Med. 136:104757. doi: 10.1016/j.compbiomed.2021.104757, PMID: 34416570

[ref16] Jackson A. F. Bolger D. J. (2014). The neurophysiological bases of EEG and EEG measurement: a review for the rest of us: neurophysiological bases of EEG. Psychophysiology 51, 1061–1071. doi: 10.1111/psyp.12283, PMID: 25039563

[ref17] Khademi Z. Ebrahimi F. Kordy H. M. (2022). A transfer learning-based CNN and LSTM hybrid deep learning model to classify motor imagery EEG signals. Comput. Biol. Med. 143:105288. doi: 10.1016/j.compbiomed.2022.105288, PMID: 35168083

[ref18] LeCun Y. Bengio Y. Hinton G. (2015). Deep learning. Nature 521, 436–444. doi: 10.1038/nature14539 26017442

[ref19] Li L.-L. Cao G.-Z. Liang H.-J. Chen J.-C. Zhang Y.-P. (2022). “EEG generation of virtual channels using an improved Wasserstein generative adversarial networks” in Intelligent robotics and applications lecture notes in computer science. eds. Liu H. Yin Z. Liu L. Jiang L. Gu G. Wu X.. (Cham: Springer International Publishing)

[ref20] Liu W. Qiu J.-L. Zheng W.-L. Lu B.-L. (2022). Comparing recognition performance and robustness of multimodal deep learning models for multimodal emotion recognition. IEEE Trans. Cogn. Dev. Syst. 14, 715–729. doi: 10.1109/TCDS.2021.3071170

[ref21] Lotte F. Bougrain L. Cichocki A. Clerc M. Congedo M. Rakotomamonjy A.. (2018). A review of classification algorithms for EEG-based brain–computer interfaces: a 10 year update. J. Neural Eng. 15:031005. doi: 10.1088/1741-2552/aab2f2, PMID: 29488902

[ref22] Luong T. Pham H. Manning C. D. (2015). Effective approaches to attention-based neural machine translation. in Proceedings of the 2015 conference on empirical methods in natural language processing (Lisbon, Portugal: Association for Computational Linguistics), 1412–1421.

[ref23] Mahesh B. (2018). Machine learning algorithms – a review. Int. J. Sci. Res. 9:7.

[ref24] Martins A. F. T. Astudillo R. F. Martins A. (2016). From Softmax to Sparsemax: a sparse model of attention and multi-label classification.

[ref25] Niu Z. Zhong G. Yu H. (2021). A review on the attention mechanism of deep learning. Neurocomputing 452, 48–62. doi: 10.1016/j.neucom.2021.03.091

[ref26] Ollikainen J. O. Vauhkonen M. Karjalainen P. A. Kaipio J. P. (1999). Effects of local skull inhomogeneities on EEG source estimation. Med. Eng. Phys. 21, 143–154. doi: 10.1016/S1350-4533(99)00038-7, PMID: 10468356

[ref27] Oostenveld R. Praamstra P. (2001). The five percent electrode system for high-resolution EEG and ERP measurements. Clin. Neurophysiol. 112, 713–719. doi: 10.1016/S1388-2457(00)00527-7, PMID: 11275545

[ref28] Phan H. Lorenzen K. P. Heremans E. Chén O. Y. Tran M. C. Koch P.. (2023). L-SeqSleepNet: Whole-cycle long sequence modelling for automatic sleep staging. Available at: http://arxiv.org/abs/2301.03441 (accessed March 24, 2023). 10.1109/JBHI.2023.3303197 37552591

[ref29] Saba-Sadiya S. Alhanai T. Liu T. Ghassemi M. M. (2020). EEG Channel interpolation using deep encoder-decoder networks. Available at: http://arxiv.org/abs/2009.12244 (accessed October 10, 2022).

[ref30] Schalk G. McFarland D. J. Hinterberger T. Birbaumer N. Wolpaw J. R. (2004). BCI2000: A general-purpose brain-computer Interface (BCI) system. IEEE Trans. Biomed. Eng. 51, 1034–1043. doi: 10.1109/TBME.2004.827072, PMID: 15188875

[ref31] Song H. Kim M. Park D. Shin Y. Lee J.-G. (2022). Learning from Noisy labels with deep neural networks: a survey. Available at: http://arxiv.org/abs/2007.08199 (accessed June 11, 2023). 10.1109/TNNLS.2022.3152527 35254993

[ref32] Soong A. C. K. Lind J. C. Shaw G. R. Koles Z. J. (1993). Systematic comparisons of interpolation techniques in topographic brain mapping. Electroencephalogr. Clin. Neurophysiol. 87, 185–195. doi: 10.1016/0013-4694(93)90018-Q, PMID: 7691549

[ref33] Soufineyestani M. Dowling D. Khan A. (2020). Electroencephalography (EEG) technology applications and available devices. Appl. Sci. 10:7453. doi: 10.3390/app10217453

[ref34] Stober S. Sternin A. Owen A. M. Grahn J. A. (2015). Towards music imagery information retrieval: Introducing the OPENMIIR dataset of EEG recordings from music perception and imagination.

[ref35] Sun H. Li C. Zhang H. (2023). Design of virtual BCI channels based on informer. Front. Hum. Neurosci. 17:1150316. doi: 10.3389/fnhum.2023.1150316, PMID: 37169016 PMC10165084

[ref36] Svantesson M. Olausson H. Eklund A. Thordstein M. (2021). Virtual EEG-electrodes: convolutional neural networks as a method for upsampling or restoring channels. J. Neurosci. Methods 355:109126. doi: 10.1016/j.jneumeth.2021.109126 33711358

[ref37] Vaswani A. Shazeer N. Parmar N. Uszkoreit J. Jones L. Gomez A. N.. (2017). Attention is all you need. Available at: http://arxiv.org/abs/1706.03762 (accessed July 19, 2022).

[ref38] Wang Y. Duan W. Dong D. Ding L. Lei X. (2022). A test-retest resting, and cognitive state EEG dataset during multiple subject-driven states. Sci. Data 9:566. doi: 10.1038/s41597-022-01607-9 36100589 PMC9470564

[ref39] Wang X.-W. Nie D. Lu B.-L. (2014). Emotional state classification from EEG data using machine learning approach. Neurocomputing 129, 94–106. doi: 10.1016/j.neucom.2013.06.046

[ref40] Xu J. Sun X. Zhang Z. Zhao G. Lin J. (2019). Understanding and improving layer normalization. Adv. Neural Inf. Process. Syst. 32.

[ref41] Xue B. Lv Z. Xue J. (2020). Feature transfer learning in EEG-based emotion recognition. In 2020 Chinese Automation Congress (CAC) (Shanghai, China: IEEE).

[ref42] Yin Z. Shen Y. (2018). On the dimensionality of word embedding. Adv. Neural Inf. Proces. Syst. 31

[ref43] Zaitcev A. Cook D. G. Liu D. W. Paley M. Milne D. E. (2017). EEG source imaging for improved control BCI performance.

[ref44] Zeng H. Yang C. Dai G. Qin F. Zhang J. Kong W. (2018). EEG classification of driver mental states by deep learning. Cogn. Neurodyn. 12, 597–606. doi: 10.1007/s11571-018-9496-y, PMID: 30483367 PMC6233328

[ref45] Zhang B. Sennrich R. (2019). Root mean square layer normalization. Adv. Neural Inf. Proces. Syst. 32

[ref46] Zheng W.-L. Liu W. Lu Y. Lu B.-L. Cichocki A. (2019). EmotionMeter: a multimodal framework for recognizing human emotions. IEEE Trans. Cybernet. 49, 1110–1122. doi: 10.1109/TCYB.2018.2797176, PMID: 29994384

[ref47] Zhong P. Wang D. Miao C. (2022). EEG-based emotion recognition using regularized graph neural networks. IEEE Trans. Affect. Comput. 13, 1290–1301. doi: 10.1109/TAFFC.2020.2994159

[ref48] Zhou H. Zhang S. Peng J. Zhang S. Li J. Xiong H.. (2021). Informer: beyond efficient transformer for long sequence time-series forecasting. AAAI 35, 11106–11115. doi: 10.1609/aaai.v35i12.17325

